# Revisiting the cardiometabolic relevance of serum amylase

**DOI:** 10.1186/1756-0500-4-419

**Published:** 2011-10-18

**Authors:** Kei Nakajima, Toshitaka Muneyuki, Hiromi Munakata, Masafumi Kakei

**Affiliations:** 1 Division of Clinical Nutrition, Department of Medical Dietetics, Faculty of Pharmaceutical Sciences, Josai University, 1-1 Keyakidai, Sakado, Saitama, 350-0295, Japan; 2 First Department of Comprehensive Medicine, Saitama Medical Center, Jichi Medical University School of Medicine, 1-847 Amanuma, Omiya, Saitama 330-8503, Japan; 3 Department of Internal Medicine, Social Insurance Omiya General Hospital, 453 Bonsai, Kita, Saitama, 331-0805, Japan

## Abstract

**Background:**

The pancreas has dual functions as a digestive organ and as an endocrine organ, by secreting digestive enzymes and endocrine hormones. Some early studies have revealed that serum amylase levels are lower in individuals with chronic pancreatitis, severe long-term type 2 diabetes or type 1 diabetes. Regarding this issue, we recently reported that low serum amylase levels were associated with metabolic syndrome and diabetes in asymptomatic adults. In the light of this, we further investigated the fundamental relationship between serum amylase and cardiometabolic aspects by reanalyzing previous data which comprised subjects without diabetes treatment with oral hypoglycemic drugs or insulin (n = 2,344).

**Findings:**

Serum amylase was inversely correlated with body mass index independently of age. Higher serum amylase levels were noted in older subjects aged 55 years old or more (n = 1,114) than in younger subjects (*P *< 0.0001, ANOVA), probably due to lower kidney function. It was likely that serum amylase may act similarly to other cardiometabolic protective factors such as high-density lipoprotein cholesterol. However, serum amylase levels were significantly lower in drinkers, particularly daily drinkers (n = 746, *P *< 0.0001, ANOVA). Meanwhile, despite of consistent inverse relationship between serum amylase and fasting plasma glucose, the relationship between serum amylase and HbA1c may be rather complicated in individuals with normal or mildly impaired glucose metabolism (up to HbA1c 6.0% (NGSP)).

**Conclusions:**

Revisiting the cardiometabolic relevance of serum amylase may yield novel insight not only into glucose homeostasis and metabolic abnormalities related to obesity, but also possibly carbohydrate absorption in the gut.

## Introduction

In recent years, many studies have provided evidence that digestive organs contribute to the control of energy balance and glucose homeostasis via gut hormones [1]. The pancreas has dual functions as a digestive organ and as an endocrine organ, by secreting digestive enzymes including amylase and endocrine hormones including insulin. The exocrine-endocrine relationship in the pancreas has been a focus of much attention in animal and cellular studies [2]. On the other hand, few clinical studies have been conducted and the clinical relevance of low serum amylase levels remains unknown, although the impact of high serum amylase levels has been investigated by numerous clinical researchers in terms of acute pancreatitis.

Some early studies have revealed that serum amylase levels are lower in individuals with chronic pancreatitis, severe long-term type 2 diabetes or type 1 diabetes [3-6], which are often accompanied by atrophic pancreas tissue. However, the associations between serum amylase levels with lifestyle factors and cardiometabolic factors remain poorly understood. Recently we reported that low serum amylase levels were associated with metabolic syndrome and diabetes in asymptomatic adults [7]. Furthermore, serum amylase levels were lower in smokers, overweight/obese subjects, and those with a greater number of metabolic syndrome components, compared with each counterpart. We describe other curious findings regarding the fundamental relationship between serum amylase and cardiometabolic aspects by reanalyzing the data used in the previous study. Animal studies have shown that insulin and a peroxisome proliferator-activated receptor-γ agonist improved the secretion of amylase [8-10]. Therefore, this time, we excluded subjects who answered to the questioner (medications for diabetes) and who had been treated with oral hypoglycemic drugs or insulin to rule out the possible influence of medications on serum amylase and to better evaluate the relationship between serum amylase and cardiometabolic risk factors including obesity and abnormal glucose metabolism.

## Methods

As described previously [7], the protocol was approved by The Ethics Committee of Josai University, and informed consent was obtained from all participants. The subjects in this study were asymptomatic Japanese aged 30-80 years who underwent thorough medical checkups at Social Insurance Omiya General Hospital, Saitama, Japan.

Subjects with C-reactive protein ≥ 10.0 mg/l, estimated glomerular filtration rate (eGFR) ≤ 35 ml/min/1.73 m^2^, serum amylase ≤ 30 IU/l or ≥ 200 IU/l, and those suspected of having cancer or endocrinopathies were excluded from the study. Furthermore, subjects who had been treated with oral hypoglycemic drugs or insulin (n = 81, details were unknown) were also excluded. Consequently, a total of 2,344 individuals were included for the present analysis.

Anthropometric and laboratory measurements were conducted with standard methods as described previously [7]. Briefly, the serum amylase level was measured using an enzymatic method (L-type Amylase, Wako, Tokyo, Japan) with a normal range of 41-112 IU/l, a detection limit of 1.7 IU/l, and a run-to-run coefficient of variation < 5.0%. Plasma glucose and HbA1c were measured by the glucose oxidase method and by high-performance liquid chromatography, respectively.

## Statistical analysis

The data are expressed as means ± SE. Serum amylase and high-density lipoprotein cholesterol (HDL-C) were examined with one-way or two-way analysis of variance (ANOVA) with six body mass index (BMI) categories as one factor, and age or drinking alcohol as another factor. Linear correlation was examined with Pearson's correlation. Statistical analysis was performed using SPSS software version 18.0 (SPSS-IBM Chicago, IL). Values of p < 0.05 were considered statistically significant.

## Findings and Discussion

**Figure 1:**
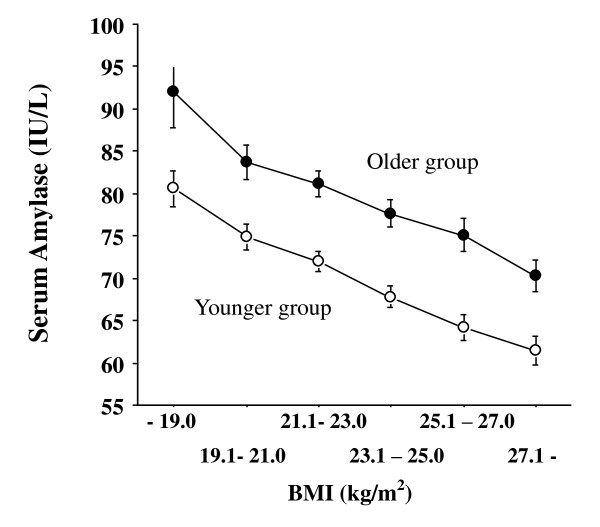
**Relationship between serum amylase and BMI according to age groups (open circle: younger subjects aged 54 years old or less, closed circle: older subjects aged 55 years old or more)**. Statistical difference was examined by two-way ANOVA and *P *value was significant at *P *< 0.0001. The numbers of subjects in six BMI groups (- 19.0, 19.1-21.0, 21.1-23.0, 23.1-25.0, 25.1-27.0, and 27.1-kg/m^2^) are 157, 371, 638, 566, 330, and 282, respectively. BMI: body mass index. Bars express standard errors.

Of cardiometabolic risk factors, high BMI, i.e., overweight/obesity, is most likely to be associated with low serum amylase independently of age, as shown in Figure 1, with an almost linear correlation between serum amylase and BMI (overall correlation, r = -0.22, *P *< 0.0001, Pearson's correlation). Partial correlation analysis controlling for age showed similar significant correlation (r = -0.21, *P *< 0.0001, data not shown). Thus, our subsequent analysis takes into account BMI. Noteworthy, serum amylase levels were significantly higher in older subjects aged 55 years old or more (n = 1,114) than in younger subjects aged 54 years old or less (*P *< 0.0001, ANOVA), probably due to lower kidney function in older subjects (serum creatinine based estimated glomerular filtration rate for older group and younger group, 71.1 ± 13.0 and 80.5 ± 12.9 ml/min/1.73 m^2^, respectively, *P *< 0.0001, t-test).

**Figure 2:**
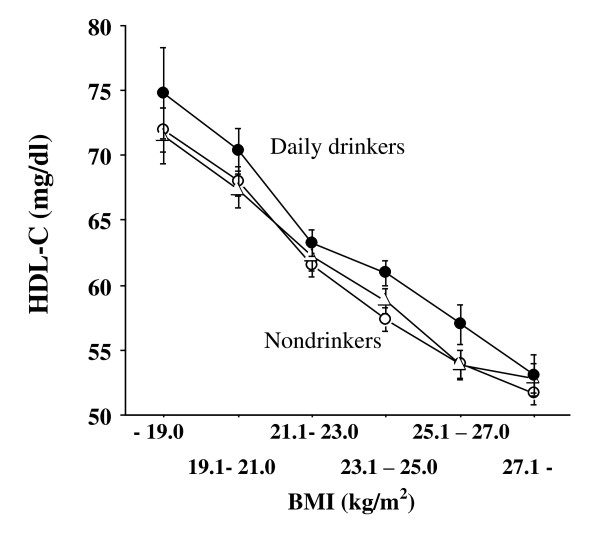
**Relationship between serum HDL-C and BMI according to alcohol consumption (open circle: nondrinkers, closed triangle: occasional drinkers, closed circle: daily drinkers)**. Statistical difference was examined by two-way ANOVA and *P *value was significant at *P *= 0.006. BMI: body mass index, HDL-C: high-density lipoprotein cholesterol. Bars express standard errors.

**Figure 3:**
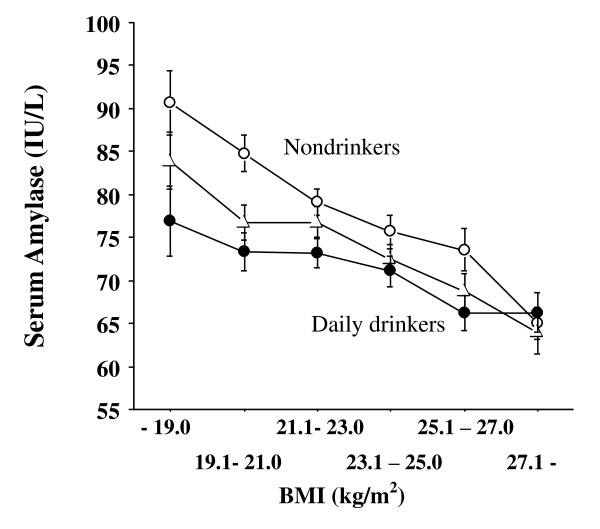
**Relationship between serum amylase and BMI according to alcohol consumption (open circle: nondrinkers, closed triangle: occasional drinkers, closed circle: daily drinkers)**. Statistical difference was examined by two-way ANOVA and *P *value was significant at *P *< 0.0001. BMI: body mass index. Bars express standard errors.

Considering these inverse associations between serum amylase and unfavorable factors such as obesity and smoking, it is likely that serum amylase may act similarly to other cardiometabolic protective factors such as HDL and adiponectin, both of which reflect better insulin sensitivity, anti-atherogenic and anti-inflammatory properties. Studies have shown that HDL-C and adiponectin levels are often elevated in mild to moderate drinkers [11]. Actually, in our study, HDL-C levels were significantly higher in drinkers (Figure 2, *P *= 0.006, ANOVA). Of note, however, is that serum amylase levels were significantly lower in drinkers, particularly daily drinkers (n = 746, Figure 3, *P *< 0.0001, ANOVA). This may be the most remarkable difference regarding alcohol consumption between serum amylase and HDL-C and adiponectin. Indeed, chronic mild to moderate alcohol consumption often injures and attenuates pancreatic functions [12,13], eventually resulting in chronic pancreatitis and reduced amylase secretion. In contrast, it has been reported that high alcohol consumption increases the risk of developing type 2 diabetes [14], which may be related to low serum amylase. However, the mechanism underlying this link between alcohol consumption and low serum amylase is unclear.

**Figure 4:**
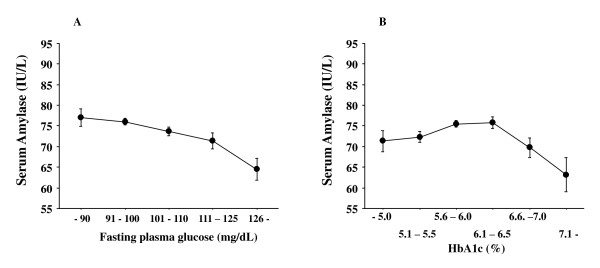
**Relationship between serum amylase and fasting glucose (A), and HbA1c (NGSP) (B)**. FPG of 90 mg/dl corresponds with HbA1c of 5.0% (NGSP), as determined by linear regression analysis between FPG and HbA1c (y = 0.02x + 3.17, where y is HbA1c and x is FPG; R^2 ^= 0.44, P < 0.0001). The numbers of subjects in six BMI groups are the same as those in Figure 1. Statistical associations were examined by one-way ANOVA and *P *value is significant at *P *< 0.0001 (A). Post hoc analysis with Bonferroni method showed that no significant difference in serum amylase was observed between any groups divided with HbA1c (B). BMI: body mass index, FPG: fasting plasma glucose, NGSP: National Glycohemoglobin Standardization Program. Bars express standard errors.

Our previous study also showed a significant association between low serum amylase and diabetes, presumably type 2 diabetes [7]. Nevertheless, the relationship between serum amylase and HbA1c, which reflects overall glucose metabolism, may be rather complicated. As shown in Figure 4A, we observed a significant but weak correlation between serum amylase and fasting glucose levels (r = -0.14, *P *< 0.0001, Pearson's correlation), but not with HbA1c (r = -0.03, *P *= 0.12) that is estimated as NGSP equivalent value calculated by the formula HbA1c (%) = HbA1c (JDS)(%) + 0.4%, considering the relational expression of HbA1c (JDS)(%) measured by the previous Japanese standard substance and measurement methods and HbA1c [15] (Figure 4B), suggesting that overall glucose homeostasis is unlikely to be associated with serum amylase, particularly in individuals with normal or mildly impaired glucose metabolism (up to HbA1c 6.0% (NGSP)). A plausible explanation is that mild insulin resistance was almost compensated with hyperinsulinemia, resulting in euglycemia (normal HbA1c) and relatively lower serum amylase levels than expected based on BMI. Moreover, glomerular hyperfiltration, which is often observed in obese people [16,17] or at early stage of diabetic nephropathy [18], might interfere with the observed associations by excess elimination of circulating amylase from the kidney. In contrast, moderate to severe insulin resistance was not compensated with hyperinsulinemia (or the hypoinsulinemia was already apparent), resulting in substantially impaired insulin action, overall hyperglycemia (high HbA1c), and very low serum amylase levels.

Other reason for the discrepancy between serum amylase and HbA1c may be that HbA1c reflects glucose metabolism over the preceding 1-2 months, whereas serum amylase may change rapidly in response to changes in plasma glucose concentration [19]. Zini et al. [10] reported that low circulating amylase normalized within 1 week of insulin therapy in cats with diabetes. Furthermore, histological examination in their study revealed that low amylase activities in diabetic cats may reflect an imbalance in glucose metabolism rather than pancreatitis. Nevertheless, further studies are needed to elucidate the mechanisms involved in the association.

In terms of the inverse association between serum amylase and obesity, some explanations are possible. Reduced insulin action including insulin resistance is a critical physiological feature accompanied with excess fat accumulation, primarily in adipose tissues. In this context, reduced pancreatic amylase secretion might represent a physiological mechanism designed to reduce the absorption of carbohydrates by suppressing their digestion, leading to reduced energy intake. Thus, it is possible that the pancreas might contribute to the overall control of energy balance and glucose homeostasis not only via its established endocrine effects, but also via the exocrine system by controlling energy intake through the small intestine.

There is a growing body of research focusing on products that slow carbohydrate absorption by inhibition of α-amylase, which is responsible for their digestion [20,21]. Many clinical studies have shown that α-amylase inhibitors (although not clinically available), most of which are extracted from plants, can reduce the post-prandial glucose excursions, thus correcting abnormal glucose metabolism and prevention of type 2 diabetes [20,21].

Meanwhile, several studies have reported the possibility that alpha-amylase is produced by some nonpancreatic gastrointestinal organs such as stomach [22,23], although controversy still remains. Therefore, low serum amylase might reflect the endocrine-gastrointestinal system, rather than the endocrine-exocrine system in the pancreas. Nevertheless, to our knowledge, no studies have examined the expression of α-amylase in the intestine or the secretion of α-amylase from the intestine in patients with type 2 diabetes. If expression or secretion of α-amylase in the gut is actually reduced in patients with type 2 diabetes, it is likely that α-amylase inhibitors, once approved, and acarbose, which inhibits both α-glucosidases and pancreatic α-amylase, should be given to people before the reductions in pancreatic amylase occur.

Regarding the possible role of serum amylase in clinical practice, collectively, low serum amylase may reflect the pathophysiological condition of obesity, a fundamental cause of MetS, which is mostly caused by energy imbalance between food intake and physical activities. On the other hand, high serum amylase may reflect thinness, i.e., possible energy shortage, and kidney dysfunction.

In conclusion, revisiting the cardiometabolic relevance of serum amylase may yield novel insight not only into glucose homeostasis and metabolic abnormalities related to obesity, but also carbohydrate absorption in the gut. Nevertheless, this hypothesis remains to be tested in future clinical and animal studies considering insulin resistance and energy imbalance.

## Competing interests

The authors declare that they have no competing interests.

## Authors' contributions

KN and HM designed the study; KN and TM reanalyzed the data; KN, HM and MK evaluated the literature and discussed; and KN wrote the first draft of the manuscript. All authors reviewed and edited the manuscript, and approved the final version of the manuscript.
